# The Combined Effects of Environmental Conditioning and Sustained Load on Mechanical Properties of Wet Lay-Up Fiber Reinforced Polymer

**DOI:** 10.3390/polym9070244

**Published:** 2017-06-23

**Authors:** Shan Li, Jiyue Hu, Huitao Ren

**Affiliations:** 1 The School of Civil Engineering, Wuhan University, Wuhan 430072, China; lishan@whu.edu.cn (S.L.); hujiyue123@foxmail.com (J.H.); 2 The School of Civil Engineering, Dalian University of Technology, Dalian 116024, China

**Keywords:** fiber reinforced polymer, durability, environmental exposure, sustained load, mechanical properties

## Abstract

The aim of this study was to investigate the combined effects of an aggressive environment and sustained load on the mechanical properties of wet lay-up fiber reinforced polymers (FRP). A total of 390 specimens, including 234 carbon fiber reinforced polymer (CFRP) specimens and 156 glass fiber reinforced polymer (GFRP) specimens, were exposed to freeze–thaw cycles, hygrothermal aging, and wet–dry cycles either in an unstressed state or loaded to about 30% or 60% of the initial ultimate load. Uniaxial tension tests were conducted on the samples after specific exposure time as well as on the control samples; and tensile properties were measured for each specimen. The results showed that the three environmental exposures, particularly hygrothermal aging, led to a significant decrease in tensile strength and elongation of the CFRP and GFRP specimens even for relatively short conditioning periods, and this decrease was markedly exacerbated by higher external loading levels. It was interesting to observe that the tensile modulus of the CFRP and GFRP specimens exhibited an excellent resistance and even appeared to increase slightly after exposure. Finally, predictive values of tensile strength based on the Arrhenius method were compared with the design values of ACI 440.2R-08 and GB 50608-2010. The results showed that both ACI 440.2R-08 and GB 50608-2010 were too conservative and significantly underestimated the tensile strength of FRP materials after an anticipated exposure period.

## 1. Introduction

In recent decades, fiber reinforced polymer (FRP) composites have been increasingly used through the wet lay-up process for strengthening deteriorated concrete structures, mainly due to their attractive properties, including excellent tensile strength, light weight, resistance to electrochemical corrosion, and ease of tailoring [1,2,3]. Although the viability of reinforced concrete structures strengthened with FRP through the wet lay-up process has been extensively demonstrated through laboratory tests and field applications, many significant unanswered questions remain regarding its durability and the extent of service life that can be expected. With regard to application, it is crucial for a designer to consider not only the short-term characteristics of the materials, but also the rates of deterioration of FRP composites as a function of exposure condition and time. 

FRP materials have been used successfully in the aerospace, automotive, and naval fields for many years, and have been verified as to their excellent long-term performance. However, the surroundings, the overall loads, the fabricating methods, and quality control in civil engineering projects are quite different from those in the aerospace or defense industry. In addition, aerospace adhesives are specially made and are superior to the structural adhesives used in the civil engineering field [4]. Thus, the durability of FRP composites in unique civil engineering environments requires investigation. 

On this issue, a number of investigations have been conducted to study the long-term performance of wet lay-up FRP composites in typical civil engineering environments, including freeze–thaw cycles, hygrothermal aging, and wet–dry cycles. Freeze–thaw exposure was identified as the most severe temperature-related threat by the Civil Engineering Research Foundation (CERF) [5], and the degradation tends to be exacerbated by moisture absorption [6,7,8]. Karbhari, Li, Rivera et al. [9,10,11,12,13,14] proceeded with freeze–thaw cycles in several typical mediums—dry air, humid air, deionized water, and salty water. With the exception of Reference [9], the overall results showed a great loss in the mechanical properties and integrity of FRP due to fiber/matrix debonding and matrix microcracking. The freeze–thaw medium type affects moisture sorption evidently, and decides how much moisture ingress degrades the composites accordingly. Herein, freeze–thaw exposure in salty water did the most deterioration, followed by deionized water, humid air, and dry air. For various hygrothermal conditions, the temperature dependence of the equilibrium water uptake makes the hygrothermal aging more aggressive than only moisture attack [15,16]. Hygrothermal durability tests conducted by Springer et al. [17] illustrated that the strength and modulus changes were decided based upon the material, the temperature, and the environment (relative humidity of air, or of liquid used). Abanilla et al. [18] presented a detailed durability investigation of CFRP immersed in deionized water at elevated temperatures, and showed the strength significantly degraded while modulus was relatively less affected. Based on these works, Xu et al. [19] summarized that the hygrothermal exposure was the most severe environmental condition to degrade the performance of polymeric materials. Xu and Miriyala et al. [19,20] also concluded that hygrothermal exposure could significantly accelerate the moisture-induced deterioration process of FRP materials and the polymer relaxation process through a series of gravimetric experiments and numerical modelling. As water desorption has been evidenced to be more destructive than absorption, therefore, the wet–dry cycle is also a typical threat to the durability of FRP for its repeated absorption and desorption process. Existing studies have demonstrated that the wet–dry cycle could decrease the ultimate load of various FRP composites [21,22]. Another study affirmed the damage of the wet–dry cycle on the strength of FRP, and stressed that this damage could be alleviated by using an inorganic matrix with the correct silica/alumina ratio [23]. Like the freeze–thaw condition, exposure results vary from different wet–dry mediums. Hulatt et al. [24] exposed the CFRP and GFRP specimens to a wet–dry cycle in the media of tap water and a salt solution. It was found that the ultimate failure stress decreased in tap water, but increased in the salt solution. Moreover, GFRP exhibited greater vulnerability than CFRP due to the etching reactions and the resulting dissolution of glass fibers [25].

In service conditions, FRP composites are simultaneously subjected to aggressive environmental conditioning and sustained loads. Composite creep under sustained load plays an important role, as it can compromise the reliability and durability of structural elements [26]. Therefore, studying the combined effects of environmental conditioning and sustained loads appears imperative and even dominates the overall response in most cases [27]. Helbling et al. [28,29] reported that sustained bending strain had no influence on moisture uptake, but could cause severe fiber/matrix interfacial debonding, fiber pitting, and cracking; their results also emphasized that the E-glass fibers were especially vulnerable when the moisture degradation and stress ruptures occur simultaneously. Kafodya et al. [30] reported that both the immersion medium (distilled water and seawater) and the degree of bending strain by 50% showed slight effects on the tensile strength, and explained that the effect of bending strain was overshadowed by the post-curing of the matrix. Shi et al. [31] reported the experimental results of the freeze–thaw resistance of FRP composites, where sustained loading was included in the testing. The results showed that sustained loading could cause further deterioration to the tensile strength of FRP composites. Therefore, the coupled actions of harsh environments and sustained load cannot be neglected when a designer assesses the durability of FRP composites.

This study presents the experimental results of the tensile properties of FRP specimens exposed to freeze–thaw cycles, hygrothermal aging, and wet–dry cycles either in an unstressed state or loaded to 30% or 60% of the ultimate load. Furthermore, the individual and coupled effects of different environments and sustained stresses on the mechanical properties of the FRP specimens were quantified. In our study, predictive equations using the Arrhenius method were also proposed to calculate the tensile strength of FRP materials after their anticipated service life. By comparing the predictive values with the design values of ACI 440.2R-08 and GB 50608-2010, the reasonability of the allowable strength design was discussed.

## 2. Experimental Program

### 2.1. Experimental Materials and Specimens

In this study, two kinds of fiber/epoxy composites—prepregs of carbon fiber reinforcement polymer (CFRP) and glass fiber reinforcement polymer (GFRP)—were used. Standard tensile coupons tests (as per ASTM D3039) were conducted to measure the material properties of CFRP and GFRP sheets, and six identical specimens per test condition were tested. A summary of the averages (AVG), and coefficients of variation (COV) is presented in Table 1. Epoxy resins I and II both consisted of two parts: Resin A and hardener B, but with different mix ratios being 3:1 and 2.5:1 by weight, respectively. Note that both epoxy resins had a pot life of 45 min and full cure time of seven days at 25 °C.

**Figure 1:**
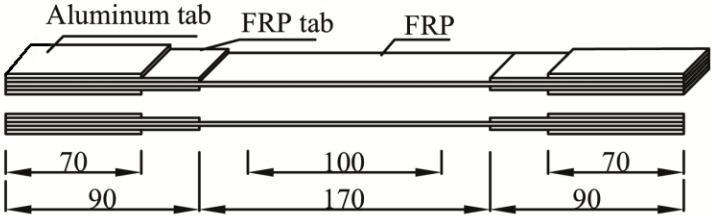
Details of FRP specimen.

Figure 1 depicts the geometry of tested specimens made in accordance with Chinese standard CECS146. First, the freshly mixed epoxy was brushed onto the surface of unidirectional fiber sheets using the wet lay-up process. Next, the specimens were fully cured for seven days at ambient temperature to solidify the epoxy resin prior to being cut into 15 mm wide × 350 mm long (CFRP) and 14 mm wide × 350 mm long (GFRP) sections. FRP strengthening tabs were then applied at the two ends of the specimens. To minimize stress concentrations near the gripping region, additional aluminum tabs were bonded at both ends of the FRP specimens. The gauge length was 100 mm.

To discover the degradation mechanisms of wet lay-up fiber-reinforced polymer under the actions of harsh environment and sustained load, a total of 390 specimens were designed, including 234 CFRP specimens and 156 GFRP specimens. The variables considered in the test were exposure condition, duration, and loading level. Table 2 lists the details of the experimental program. Six values for each regime were averaged for the sake of error reduction.

### 2.2. Sustained Load 

**Figure 2:**
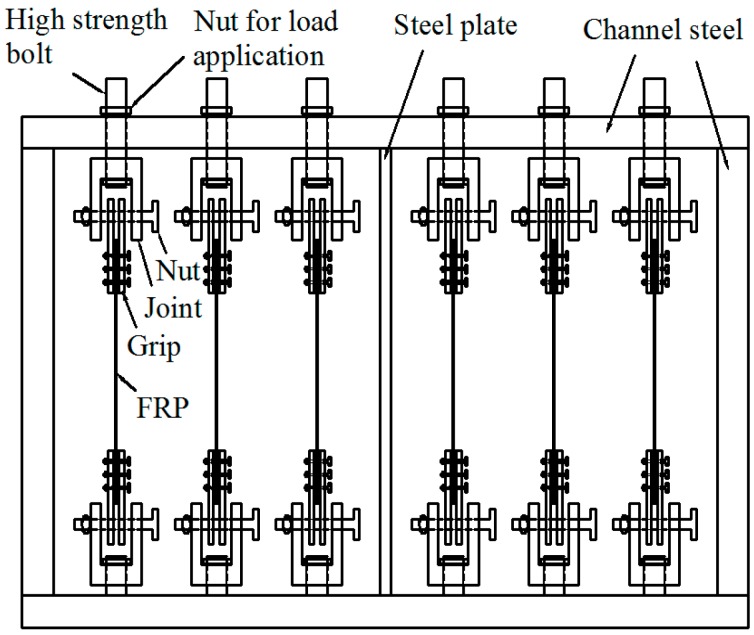
Schematic diagram of sustained loading apparatus.

Imposing a sustained tensile load on the specimens was of much significance considering the in-service conditions. A specially-designed and self-made frame was used to provide a sustained load (Figure 2), and consisted of three parts: Load carrying boards, grips, and connectors. Every six specimens suffered a sustained load in one frame. The integral operations abided by the following two steps:

1. The exact loading value was determined in advance. To avoid creep rupture of the FRP materials, the sustained stress was limited by ACI 440.2R-08 [32]. Considering the creep limits for the FRP materials, 30% and 60% of the ultimate load for CFRP (corresponding strains were 618 με and 1236 με, respectively), 30% of ultimate load for GFRP (corresponding strain was 915 με) were used in this study.
2. The sustained load was applied by tightening nuts. However, a sustained load cannot be kept unchanged due to the creep or relaxation of the FRP materials. Therefore, to assess the effects of creep or relaxation on the applied load, the strains of FRP specimens were monitored using a self-made arched strain clamp within 10 days. The results indicated that the applied load fell off measurably, but not excessively (varying between 1% and 3% of their ultimate load).

### 2.3. Environmental Exposure

The experimental regime was divided into three different environmental conditions: Freeze–thaw cycles, hygrothermal aging, and wet–dry cycles.

GB/T 50082-2009 [33] was followed appropriately in our experimental protocol for testing the FRP specimens subjected to freeze–thaw cycles. The freeze–thaw exposure was processed between −17 ± 2 °C and +8 ± 2 °C at the rate of one cycle per day in fresh water. For reference purposes, 50, 100, 200, and 300 freeze–thaw cycles were considered.

A hygrothermal environment was simulated by a salt spray tester (Wuxi Sunan Experimental Equipment Corporation, Wuxi, China) in accordance with GB/T 2574-2008 [34]. Storing the specimens in the tester for 30, 90, 180, 360 days, where high temperature (50 ± 2 °C) and high humidity (93 ± 3% RH) conditions were set. During the test process, a thermometer and a hygrometer were additionally placed to verify the pre-set temperature and humidity.

To simulate in-site conditions of offshore wet–dry exposure, the specimens were fully immersed in a bucket filled with 5% NaCl simulated seawater for 12 h before being subjected to accelerated blow-drying for another 12 h per cycle. For reference, 30, 90, 180, and 360 wet–dry cycles were considered.

Once the predetermined exposure cycles were accomplished, the specimens were placed in the lab to air dry until water desorption had concluded.

### 2.4. Tensile Test

**Figure 3:**
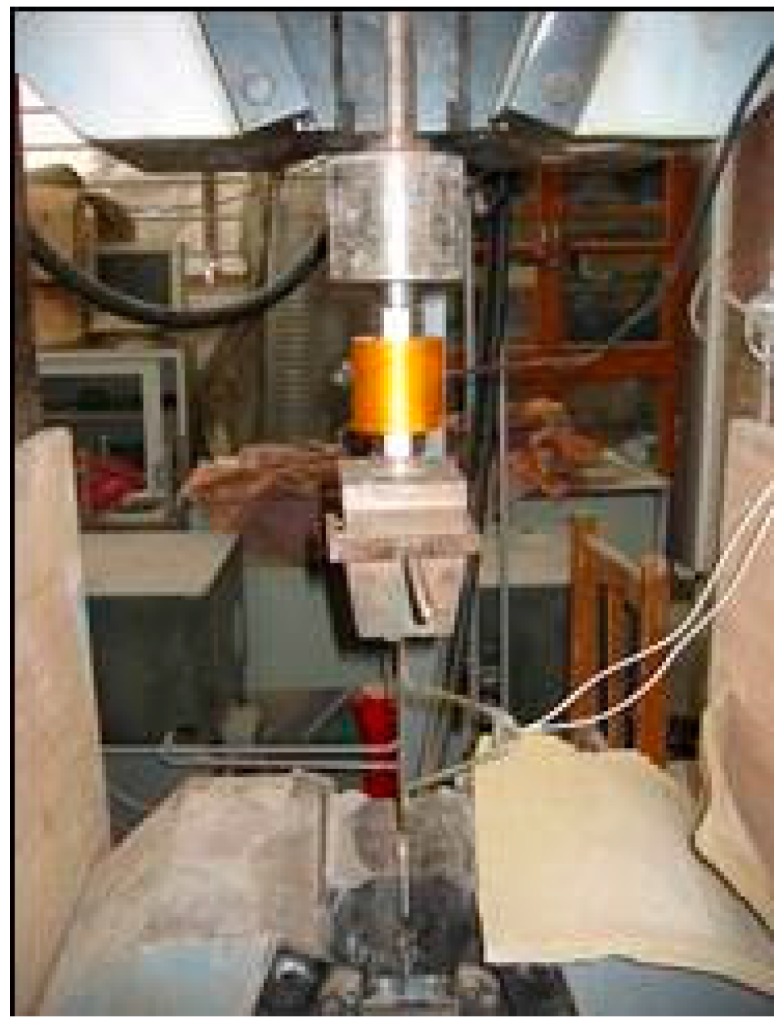
Practical test setup.

All specimens were removed from the sustained loading setup and harsh environments after a specified period of exposure, then tested for tensile properties in accordance with ASTM D3039. The test was conducted using an electro-hydraulic servo universal testing machine (Jinan Shijin Group Corporation, Jinan, China) at a speed of 1 mm/min within the gauge length of 100 mm. The schematic view of test setup is shown in Figure 3. Load and strain data were recorded using an eight-channel wet–dry digital data acquisition system (IMC, Berlin, Germany) at a frequency of 100 samples/s.

## 3. Results

In this section, three basic mechanical parameters, including tensile strength, elongation, and tensile modulus, were discussed for each regime. A more detailed description of the effects of environmental condition, loading level, and exposure duration on the abovementioned mechanical parameters follows.

### 3.1. Tensile Strength

**Figure 4:**
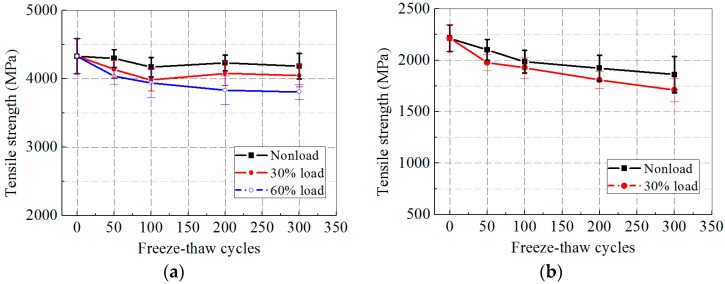
Ultimate tensile strength versus exposure time after freeze–thaw cycles: (**a**) CFRP; and (**b**) GFRP.

The changes in tensile strength of the CFRP and GFRP specimens as a function of the number of freeze–thaw cycles are presented in Figure 4a,b, respectively. A steady decrease in tensile strength of the GFRP specimens was observed as the number of freeze–thaw cycles increased. After 300 freeze–thaw cycles, the reductions in tensile strength of the CFRP specimens when compared with their initial tensile strength were 3.3%, 6.6%, and 12.0% at 0, 30%, 60% loading levels, respectively. For the GFRP specimens, the tensile strength reduced by 16.0% and 22.8% at 0 and 30% loading levels, respectively. It was deduced from these data that the increasing sustained loading level could aggravate tensile strength degradation. For the two types of FRP specimens, GFRP was more vulnerable to both freeze–thaw exposure and sustained load due to etching reactions and the resulting dissolution of the glass.

**Figure 5:**
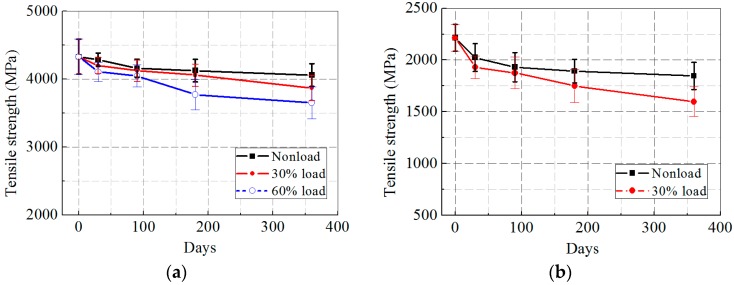
Ultimate tensile strength versus exposure time after hygrothermal aging: (**a**) CFRP; and (**b**) GFRP.

Figure 5a,b illustrates the tensile strength of the CFRP and GFRP specimens after the synergetic actions of hygrothermal aging and sustained load. In contrast to the freeze–thaw cases, both CFRP and GFRP specimens performed a continuous deterioration in tensile strength, which was faster at a higher loading level. For the CFRP specimens, the tensile strength deceased by 6.3%, 10.6%, and 15.7% in 0, 30%, and 60% loading cases, respectively, after 360 days of hygrothermal exposure, whereas for the GFRP specimens, the tensile strength dropped by 16.6% and 27.9% in 0 and 30% loading cases, respectively. Clearly, hygrothermal exposure was harmful to the tensile strength of the FRP specimens, and an increasing loading level accelerated the degradation of tensile strength significantly.

**Figure 6:**
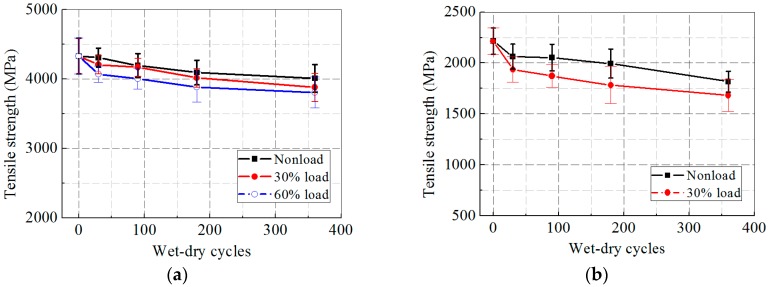
Ultimate tensile strength versus exposure time after wet–dry cycles: (**a**) CFRP; and (**b**) GFRP.

Figure 6a,b presents the tensile strength of the CFRP and GFRP specimens subjected to wet–dry cycles and sustained load. Similar to the other two exposures, the tensile strength of the FRP specimens decreased steadily with prolonged wet–dry cycles and at a higher loading level, where the tensile strength loss of the CFRP specimens was 7.5%, 10.4%, and 12.1% in 0, 30%, and 60% loading cases, respectively, whereas the drop of the GFRP specimens was 18.0% and 24.1% in 0 and 30% loading cases.

The tested data showed that the single action of environmental conditioning could degrade the tensile strength of the FRP specimens, and the combined actions of environmental conditioning and sustained load could aggravate this degradation further. This result may be due to external tensile stress exacerbating tensile strength deterioration by producing microcracks and allowing more moisture ingress; therefore, the creep effects of sustained loads are aggravated by environmental conditioning in turn [21,26,28,35,36]. For the load-free case, the reduction of tensile strength after 360 wet–dry cycles was about 7.5% and 18.0% for the CFRP and GFRP specimens, respectively. Compared with the corresponding decrease value in freeze–thaw (3.3% for CFRP and 16.0% for GFRP) and hygrothermal (6.3% for CFRP and 16.6% for GFRP) cases, the tensile strength degradation was clearly the greatest in wet–dry exposure in the load-free condition. For the 30% and 60% loading cases, the tensile strength loss of FRP specimens was the greatest in hygrothermal conditions rather than wet–dry cycles, indicating that sustained loads had greater effects on the tensile strength combined with hygrothermal aging than with the other two environmental conditions. In addition, Reference [35] pointed out that the exposed specimens exhibited more dispersion than the control specimens, and the overall scatter increased with prolonged exposure time. However, this conclusion is in conflict with the measured data in this study, where the standard deviation barely changed across all exposures. Furthermore, it must be noted that the standard deviation of the FRP specimens subjected to environmental conditioning and sustained load was slightly larger than that of the FRP specimens subjected only to environmental conditioning, which may have been due to the extra uncertainties.

### 3.2. Elongation

**Figure 7:**
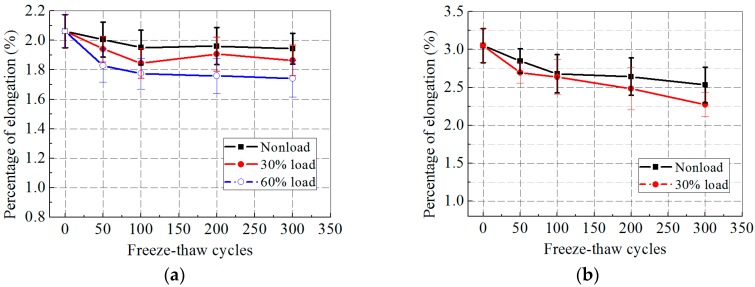
Percentage of elongation versus exposure time after freeze–thaw cycles: (**a**) CFRP; and (**b**) GFRP.

Figure 7a,b shows the elongation degradation of the CFRP and GFRP specimens with increasing freeze–thaw cycles. As can be seen, the elongation of the CFRP specimens reduced by 5.8%, 9.6%, and 15.6% at 0, 30%, and 60% loading levels after 300 freeze–thaw cycles. The drops for the GFRP specimens after the same period were 17% and 25.5% at 0 and 30% loading levels, respectively. By comparing these values with their corresponding tensile strength degradation, it was found that the elongation degradation was greater than that of the tensile strength.

**Figure 8:**
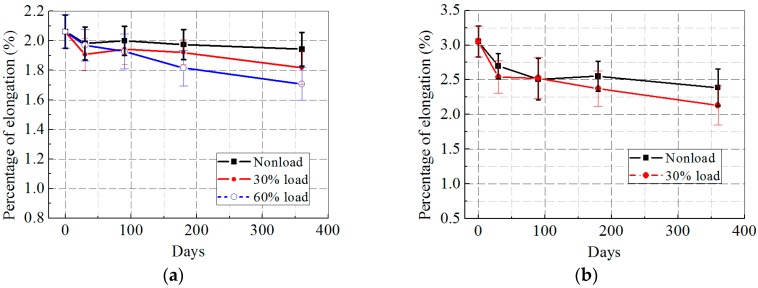
Percentage of elongation versus exposure time after hygrothermal aging: (**a**) CFRP; and (**b**) GFRP.

Figure 8a,b shows the elongation variation of the CFRP and GFRP specimens with prolonged hygrothermal time. An anomalous phenomenon occurred in the 30% loading case, which exhibited a dramatic decline in elongation of the CFRP specimens even faster than the 60% loading case in the initial 30 days. However, in the extended exposure time, elongation at 30% loading level increased in the following 60 days and finally led to a steady decrease. Moreover, it is worth noting that the hygrothermal aging did the most severe degradation at every loading level in the elongation of the GFRP specimens (reduced by 21.8% and 30.1% for 0 and 30% loading cases, respectively) compared with the other two environmental conditions. 

**Figure 9:**
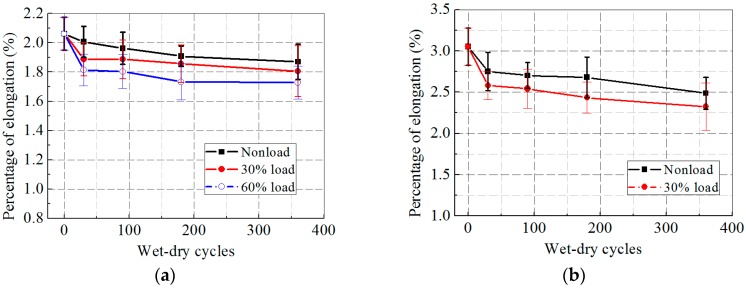
Percentage of elongation versus exposure time after wet–dry cycles: (**a**) CFRP; and (**b**) GFRP.

As shown in Figure 9a,b, a continuous decreasing elongation of the CFRP and GFRP specimens was observed with increasing wet–dry cycles and higher external loading levels. The elongation of the CFRP specimens subjected to wet–dry cycles dropped by 9.3%, 12.4%, and 16.2% for 0, 30%, 60% loading cases, where the elongation loss was the greatest at 0 and 30% loading levels when compared with freeze–thaw and hygrothermal exposures. The elongation of the GFRP specimens fell by approximately 18.5% and 23.8% at 0 and 30% loading levels.

The measured data illustrates that the environmental conditions make the CFRP and GFRP specimens embrittled and that external loads significantly enhance this embrittlement [18,37]. Furthermore, it was observed that the degradations of elongation and tensile strength after exposures were highly similar, which was verified by References [31,38,39], where a linear relationship between the reductions of elongation and tensile strength was proposed. 

### 3.3. Tensile Modulus 

**Figure 10:**
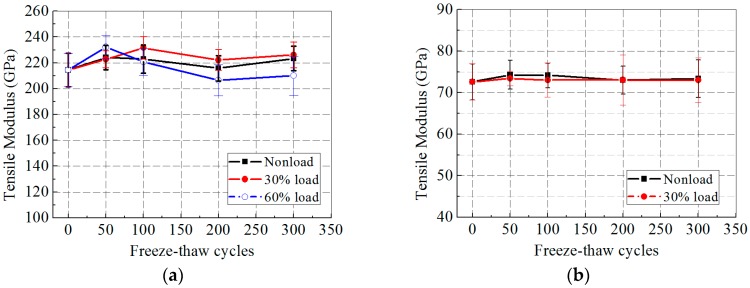
Tensile modulus versus exposure time after freeze–thaw cycles: (**a**) CFRP; and (**b**) GFRP.

Figure 10a,b presents the tensile modulus of the CFRP and GFRP specimens after freeze–thaw cycles. The tensile modulus of the GFRP specimens was barely affected by freeze–thaw exposure and sustained load, for its value increased by only about 1.1% and 0.5% at 0 and 30% loading levels. After 300 freeze–thaw cycles, the tensile modulus of the CFRP specimens increased by 4.0% and 5.4% in unstressed and 30% loading cases, but dropped by 2.1% of the control value in the 60% loading case.

**Figure 11:**
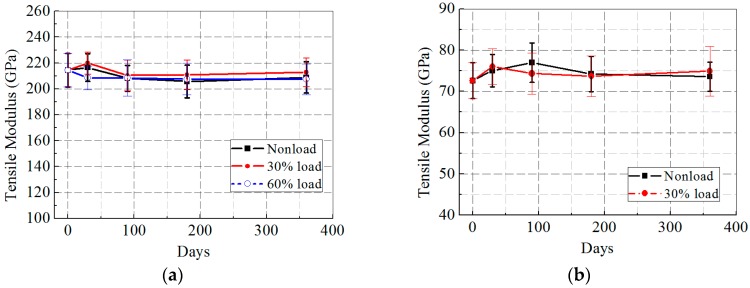
Tensile modulus versus exposure time after hygrothermal aging: (**a**) CFRP; and (**b**) GFRP.

Figure 11a,b presents the tensile modulus of the CFRP and GFRP specimens after hygrothermal aging. From Figure 11a, it can be seen that the tensile modulus of the CFRP specimens increased during the first 30 days, but decreased in the following 60 days, before finally remaining unchanged. 360 days of hygrothermal aging resulted in reductions in the tensile modulus of the CFRP specimens equivalent to approximately 2.6%, 0.7%, and 3.3% in 0, 30%, and 60% cases, respectively. As Figure 11b shows, in contrast to the freeze–thaw conditioning, the tensile modulus of the GFRP specimens changed drastically during hygrothermal aging where a marked increase of approximately 1.3% and 8.9% at 0 and 30% loading levels, respectively, in the tensile modulus of the GFRP specimens was observed after 360 days.

**Figure 12:**
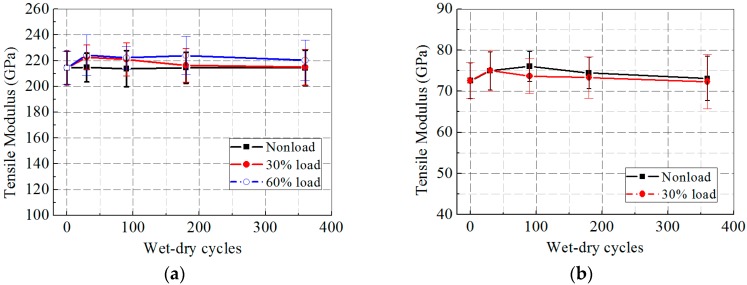
Tensile modulus versus exposure time after wet–dry cycles: (**a**) CFRP; and (**b**) GFRP.

Figure 12a,b presents the tensile modulus of the CFRP and GFRP specimens after wet–dry cycles. As seen in Figure 12a,b, the tensile modulus of the CFRP and GFRP specimens increased at first and decreased finally with exposure to wet–dry cycles. Compared with the other two environmental conditions, the wet–dry cycles only minimally influenced the tensile modulus of the FRP specimens. Apart from a reduction of 2.9% in the tensile modulus of the CFRP specimens at 60% loading level, there appeared to be no change to the tensile modulus of the CFRP and GFRP specimens after 360 wet–dry cycles.

**Figure 13:**
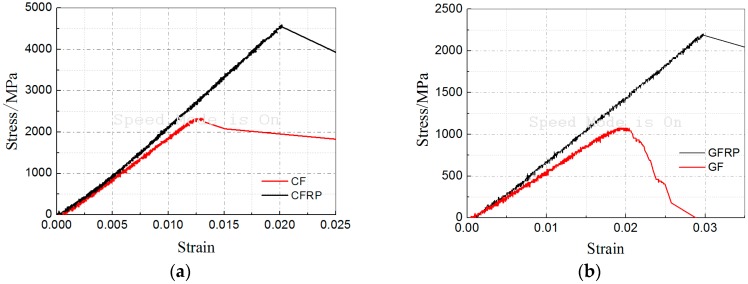
Stress-strain response: (**a**) CF (carbon fiber sheet without epoxy resin) and CFRP; (**b**) GF (glass fiber sheet without epoxy resin) and GFRP.

It was interesting to note that there was almost no apparent effect of the harsh environments and sustained loads on the tensile modulus of the FRP specimens, which was contrary to the development trends of tensile strength and elongation. The reason for the steady tensile modulus was probably due to the fact that the epoxy was susceptible to the environmental conditioning and sustained load degradations, but only occupied a very small volume fraction, which consequently resulted in the fiber-dominated tensile modulus of the composites performing almost no change in all cases. To verify this proposal, FRP (fiber sheets with epoxy resin) specimens and their respective fiber sheets without epoxy resin were tested to obtain their tensile properties. As shown in Figure 13a,b, the tensile strength and elongation of the FRP specimens were apparently larger than those of their respective fiber sheets without epoxy resin, while the tensile modulus showed little difference. From these data, it could be concluded that the presence of epoxy resin enhanced the cooperation between the independent fibers and increased the strength and deformation capacity accordingly, but contributed little to increasing the tensile modulus. In fact, there was even an increase in tensile modulus in the initial stage, which may be due to the extraction of un-cured small molecules and the resulting hardening of the epoxy [40]. Moreover, the hardening caused by molecule rearrangement with imposed sustained tensile loads may account for this increase in modulus because, in the initial stage, the increase rate of the loaded specimens performed slightly faster than that of the unloaded specimens. This discovery coincides with the experimental results described in References [13,18,24,28,29], but conflicts with the results in Reference [30], where a reduction of 16% in modulus after 90 freeze–thaw cycles was obtained.

## 4. Discussion

The effectiveness of the FRP materials during their service life of rehabilitation and strengthening is essential, so it is necessary to assess whether the tensile strength of FRP materials subjected to anticipated exposures meets the requirements of design specifications. However, it seems to be difficult to obtain the tensile strength of exposed FRP materials through a series of long-term durability tests. Therefore, the experimental results of short-term tests are often used to predict the tensile strength of the FRP materials after an anticipated exposure period. Among many predictive methods, conventional Arrhenius predictions provide good accuracy. In this section, predictive formulas of tensile strength of FRP materials versus exposure time (or cycles) were proposed in terms of the Arrhenius method. Then, the predictive values were compared with the allowable strength designs of ACI 440.2R-08 and GB 50608-2010 to assess the reasonability of the allowable design.

### 4.1. Predictive Equations

Prediction equations have already been proposed to estimate the long-term deterioration of FRP materials using the Arrhenius method, which has been proven in accuracy [14,36,41]. The equation for predicting the tensile strength of FRP materials is in the form:

(1) $$f(t)=\frac{f_{0}}{100}[A\ln(t)+B],\;\left(t>0\right)$$

where *f*(*t*) and *f*_0_ are the tensile strengths at time *t* (in days or cycles), and 0 (in the unexposed condition), respectively; *A* is a constant denoting degradation rate; and *B* is a material constant, which reflects the early effects of post-cure progression.

In this study, as mentioned earlier, the tensile strength of CFRP and GFRP specimens decreased continuously with an extended aging time. Furthermore, it has been clarified by the experimental results that the external loads would not change the degradation mechanism, but only exacerbate the tensile strength degradation of the CFRP and GFRP specimens. Therefore, Equation (1) can be used to predict the tensile strength of the CFRP and GFRP specimens subjected to environmental conditioning and sustained load. By regression analysis, equations for predicting the tensile strength of the CFRP and GFRP materials are listed in Table 3. The correlation coefficient was more than 0.78.

### 4.2. Comparison of Predictive Values with Design Values

To ensure the effectiveness of rehabilitation over the design life of the structure, it is imperative to reduce the tensile strength of FRP materials used in design equations considering long-term exposure to anticipated environments. ACI 440.2R-08 [32] restricts the design ultimate tensile strength, $f_{\mathrm{fu}}$, with an environmental reduction factor, *C*_E_, such that:

(2) $$f_{\mathrm{fu}}=C_{E}f_{\mathrm{fu}}^{*}$$

(3) $$f_{\mathrm{fu}}^{*}=(\bar{f_{\mathrm{fu}}}-3\sigma )$$

where $f_{\mathrm{fu}}^{*}$ is the ultimate tensile strength reported by manufacturers, and is defined as the mean ultimate strength of a sample of test specimens; and $\bar{f_{\mathrm{fu}}}$, minus three times the standard deviation σ. For cases of exposure in aggressive environments, *C*_E_ = 0.85 and 0.5 for the CFRP and GFRP materials, respectively.

The Chinese code GB 50608-2010 [42] suggests that the design ultimate tensile strength of FRP materials be determined by modifying the standard ultimate tensile strength, $f_{\mathrm{fk}}$, by partial safety factors:

(4) $$f_{\mathrm{fd}}=\frac{f_{\mathrm{fk}}}{\gamma_{f}\gamma_{E}}$$

(5) $$f_{\mathrm{fk}}=\mu_{f}-1.645\sigma_{f}$$

where $\gamma_{f}$ is material factor considering the reliability index and the brittle failure behavior of FRP materials. For FRP sheet and reinforcement, $\gamma_{f}=1.4$. $\gamma_{E}$ is the environmental influence factor, when exposed in aggressive environments, $\gamma_{E}=1.2$ and 1.6 for the CFRP and GFRP materials, respectively. $\mu_{f}$ and $\sigma_{f}$ are the mean ultimate strength and the standard deviation of test FRP specimens, respectively.

**Figure 14:**
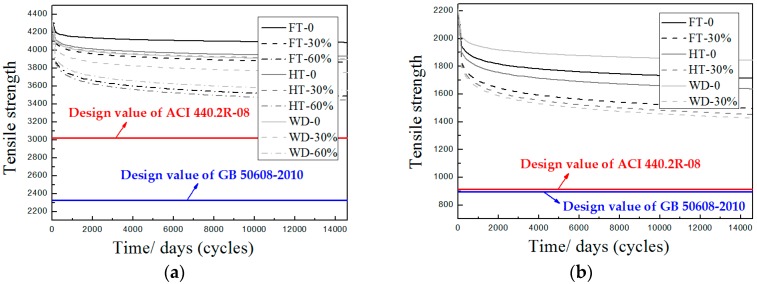
Tensile strength versus exposed time (or cycles) according to predictive equations in Table 3: (**a**) CFRP; and (**b**) GFRP.

Figure 14 depicts the tensile strength degradation versus exposure time (or cycles) as per the predictive equations in Table 3, where “FT” stands for freeze–thaw cycles, “HT” represents hygrothermal aging, and “WD” represents wet–dry cycles. Herein, the design values of ACI 440.2R-08 and GB 50608-2010 are drawn in Figure 14 in order to provide a visual comparison. As seen, there was a rapid decrease of tensile strength of FRP materials in the early stage, following a slow-speed development. Clearly, the predictive tensile strength of FRP materials was more than sufficient in terms of the two design requirements. Thus, the following discussion is directed towards the most severe cases among the different environments and loading levels. For the CFRP materials, the most severe condition was hygrothermal aging in the presence of 60% external loads. In this case, the predictive value of tensile strength of the CFRP materials was 3467.73 MPa after 10,950 days (which corresponds to 30 years) exposure, which was underestimated as much as 12.8% by ACI 440.2R-08 and 33.0% by GB 50608-2010. For the GFRP materials, wet–dry cycles accompanied by 30% external loads caused the greatest degradation to the tensile strength of the GFRP materials. After 10,950 wet–dry cycles, the predictive tensile strength of the GFRP materials was 1449.23 MPa, which was undervalued as much as 36.9% by ACI 440.2R-08 and 38.0% by GB 50608-2010. The comparative results showed that both ACI 440.2R-08 and GB 50608-2010 were too conservative, especially GB 50608-2010, and significantly underestimate the tensile strength of FRP materials, even in the context of neglecting the combined effects of environmental conditioning and sustained load. This underestimation of tensile strength may result in the inadequate utilization of FRP materials.

## 5. Conclusions

This paper describes an experiment of 234 CFRP specimens and 156 GFRP specimens subjected to the combined actions of aggressive environment and sustained load. The specimens were exposed to freeze–thaw cycles, hygrothermal aging, and wet–dry cycles under 30% and 60% ultimate loads, which simulated typical in-site conditions in civil engineering. Through a series of uniaxial tension tests, tensile strength, elongation, and tensile modulus of the FRP specimens were measured. Based on the experimental results, the following conclusions were drawn:

(1) For the load-free condition, wet–dry cycles did the most severe damage on the tensile strength of both the CFRP and GFRP specimens. However, for 30% and 60% loading conditions, the tensile strength showed the most serious degeneration after hygrothermal aging. From these results, it was reasonable to conclude that sustained loads had greater effects on the tensile strength combined with hygrothermal aging than with the other two environmental conditionings. Additionally, increasing the sustained loading level reduced the tensile strength of the CFRP and GFRP specimens significantly, indicating that external tensile stress exacerbated tensile strength deterioration by producing microcracks, thus allowing more moisture ingress. The GFRP specimens were more vulnerable than the CFRP specimens to both harsh environments and sustained loads under these exposure conditions. 

(2) A continuous decrease in the elongation of the CFRP and GFRP specimens was observed with longer exposure time and a higher loading level, which was highly similar to the degradation trend of tensile strength. The remarkable deterioration of tensile strength and elongation measured in this study proved the importance of taking the combined effects into consideration when assessing the durability of FRP materials. 

(3) As opposed to tensile strength and elongation, there was an almost negligible effect of environmental conditioning and sustained loads on the tensile modulus of the CFRP and GFRP specimens. 

(4) Predictive equations based on the Arrhenius method were proposed. The predictive values of tensile strength after an anticipated exposure period were compared with the design values based on ACI 440.2R-08 and GB 50608-2010. Both ACI 440.2R-08 and GB 50608-2010 were too conservative in the context of neglecting the combined effects of aggressive environment and sustained load, which may result in the inadequate utilization of FRP materials.

## Figures and Tables

**Table 1:** Material properties.

Material	Nominal Thickness (mm)	Young’s Modulus (GPa)		Tensile Strength (MPa)		Failure Strain (%)	
		AVG	COV (%)	AVG	COV (%)	AVG	COV (%)
CFRP sheet	0.111	214.4	6.0	4331	6.0	2.06	5.4
GFRP sheet	0.169	63.1	6.0	2138	5.8	3.53	7.4

AVG = average value; COV = coefficient of variation.

**Table 2:** Details of the experimental program.

Environmental Type	FRP Type	Loading Level	Exposure Duration/Times	Number of Specimens
Freeze–thaw cycles	CFRP	0, 30%, 60%	0, 50, 100, 200, 300 times	90
	GFRP	0, 30%		60
Hygrothermal aging	CFRP	0, 30%, 60%	0, 30, 90, 180, 360 days	90
	GFRP	0, 30%		60
Wet–dry cycles	CFRP	0, 30%, 60%	0, 30, 90, 180, 360 times	90
	GFRP	0, 30%		60

**Table 3:** Predictive equations.

Environmental Type	Loading Level	CFRP		GFRP	
		Predictive Equation	*R* ^2^	Predictive Equation	*R* ^2^
Freeze–thaw cycles	0	$f(n)=\frac{f_{0}}{100}[-0.592\ln(n)+100]$	0.802	$f(n)=\frac{f_{0}}{100}[-2.358\ln(n)+100]$	0.855
	30%	$f(n)=\frac{f_{0}}{100}[-1.128\ln(n)+100]$	0.999	$f(n)=\frac{f_{0}}{100}[-3.383\ln(n)+100]$	0.915
	60%	$f(n)=\frac{f_{0}}{100}[-2.033\ln(n)+100]$	0.974		
Hygrothermal aging	0	$f(t)=\frac{f_{0}}{100}[-0.962\ln(t)+100]$	0.953	$f(t)=\frac{f_{0}}{100}[-2.721\ln(t)+100]$	0.968
	30%	$f(t)=\frac{f_{0}}{100}[-1.038\ln(t)+100]$	0.930	$f(t)=\frac{f_{0}}{100}[-3.588\ln(t)+100]$	0.970
	60%	$f(t)=\frac{f_{0}}{100}[-2.143\ln(t)+100]$	0.780		
Wet–dry cycles	0	$f(n)=\frac{f_{0}}{100}[-1.052\ln(n)+100]$	0.838	$f(n)=\frac{f_{0}}{100}[-1.75\ln(n)+100]$	0.980
	30%	$f(n)=\frac{f_{0}}{100}[-1.406\ln(n)+100]$	0.781	$f(n)=\frac{f_{0}}{100}[-3.717\ln(n)+100]$	0.956
	60%	$f(n)=\frac{f_{0}}{100}[-1.877\ln(n)+100]$	0.942		

*R*^2^ = correlation coefficient, *t* = exposed time; and *n* = number of cycles.
